# Stability of color and biologically active compounds of pasteurized juices from potatoes with colored flesh

**DOI:** 10.1002/fsn3.4102

**Published:** 2024-06-12

**Authors:** Agnieszka Tkaczyńska, Elżbieta Rytel, Alicja Z. Kucharska, Joanna Kolniak‐Ostek, Anna Sokół‐Łętwska

**Affiliations:** ^1^ Department of Food Storage and Technology Wrocław University of Environmental and Life Sciences Wrocław Poland; ^2^ Departament of Fruit, Vegetable and Plant Nutraceutical Technology Wrocław University of Environmental and Life Sciences Wrocław Poland

**Keywords:** anthocyanin content, color stabilization, glycoalkaloid content, pasteurization, potato juices with color flesh, total polyphenol content

## Abstract

Juices from potato varieties with colored flesh contain a large amount of biologically active compounds, but they tend to darken enzymatically, which deteriorates the quality. One of the factors that can improve the color of juices is pasteurization. The aim of the study was to investigate the effect of pasteurization temperature on the anthocyanin content and color of juices from potatoes with colored flesh. The research material included juices from potato varieties with red and purple flesh. Juices pasteurized at 75 °C were characterized by the lightest color and an increase in the a* (red color) and b* (yellow color) parameters compared to unpasteurized juices. Pasteurization of juices reduced the amount of glycoalkaloids by an average of 54% compared to unpasteurized juices (larger losses in the content of α‐chaconine than α‐solanine). Purple potato juices showed a higher content of total polyphenols by an average of 30% and anthocyanins by 70% than juices from red potatoes. Pelargonidin and its derivatives were identified in red potato juices, while petunidin and peonidin were the most abundant in purple potato juices. Higher losses of total polyphenols were found in juices from red varieties of potatoes, while anthocyanins were less thermostable in juices from varieties with purple flesh.

## Graphical abstract

Pasteurization of juices reduced glycoalkaloids by 54% compared to nonpasteurized. Pasteurization influenced the composition and content of anthocyanins. Less thermostable were anthocyanins in purple potato juices.

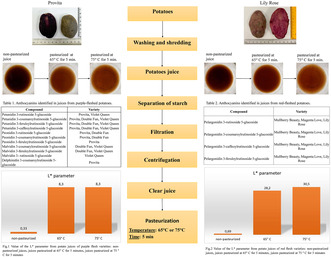

## 1 INTRODUCTION

The potato industry is one of the leading industries across European countries. Approximately 10 million tons of starch and starch products along with over 5 million tons of protein and fibers are produced annually in the European Union. This contributes to the generation of a vast amount of waste, with the starch industry alone producing over 3.5 tons of potato juice (Lubiewski et al., 2006; Zwijnenberg et al., 2002). The juice is obtained after potato pulp separation and is a by‐product of the starch production process. In most processing plants, potato juice is used to extract protein for animal feedstuffs, which is a good example of onerous waste management. However, in small processing plants, the juice left after potato pulp separation is not utilized and becomes a production waste. This results in high waste disposal costs and poses a serious environmental problem (Milner et al., 2011). Although potato juice is a by‐product of potato processing, it is still valuable due to its chemical composition as it contains all soluble compounds naturally occurring in potatoes, that is, approximately 2% crude protein, 0.5%–0.8% carbohydrates, 0.2% fat, 1% minerals, including mainly potassium (approximately 0.6%) and phosphorus (approximately 0.3%), as well as 0.15% fiber and starch (Kowalczewski et al., 2021). Vitamin C, naturally present in potatoes, is degraded during preservation and separation processes (Das et al., 2012). Due to its chemical composition, including particularly its biologically active compounds, potato juice can be classified among functional foods and nutraceuticals (Kaur & Das, 2011; Kowalczewski et al., 2022). Research conducted in numerous scientific centers worldwide has highlighted the recent trend of seeking new sources of nutraceuticals and functional food (Baranowska et al., 2018; Kowalczewski, Lewandowicz, Makowska, et al., 2015b). The latter has gained many supporters in the United States and Europe, which has led to the dynamic growth in its production and consumption. It is defined as food that exhibits a scientifically proven beneficial impact on one or more bodily functions beyond its nutritional value, improving health status, and well‐being, and/or reducing the risk of development of multiple diseases (Kim et al., 2012; Kowalczewski, Różańska, Makowska, Jeżowski, & Kubiak, 2019b). Functional food should occur in a natural or technologically modified form and should not be produced in the form of tablets or dietary supplements, like nutraceuticals. Potato juice meets these requirements as it exhibits antioxidant, anti‐inflammatory, and anticarcinogenic properties, and so far has been used solely for consumption in folk medicine (Benkeblia, 2020; Kowalczewski, Lewandowicz, Krzywdzińska‐Bartkowiak, et al., 2015a). Production of potato juice from red‐fleshed and purple‐fleshed potato cultivars may prove particularly beneficial because potatoes of these cultivars have approximately 2–3 times higher content of biologically active compounds, especially rich in phenolic acids and anthocyanins (Kowalczewski, Olejnik, Białas, Kubiak, et al., 2019c). Anthocyanins are natural pigments, imparting colors, from pink through purple to blue, to plant raw materials or foods containing them (Sun et al., 2013). The molecules present in colored potatoes are acylated with p‐coumaric or ferulic acids. Acylation of anthocyanin molecules improves pigment stability during processing, affording greater possibilities for potato utilization as a source of natural colorants compared to fruit‐derived anthocyanins (Kubow et al., 2014; Kujawska et al., 2018; Mattioli et al., 2020; Singh et al., 2020). Juices obtained from red‐fleshed and purple‐fleshed potato cultivars may differ in chemical composition, color intensity, as well as content and stability of biologically active compounds (Jansen & Flamme, 2006). In addition to the positive aspects of using potato juice for consumption or as a food additive, it has also certain drawbacks, especially due to glycoalkaloids being one of the adverse compounds naturally occurring in potato tubers. They are mainly found in the potato skin. Most of them can be removed during processing, but some may remain in the juice even after pulp separation.

One of the main factors influencing the reduction in the quantity of glycoalkaloids in potatoes is replaced by peeling. Based on research conducted by other authors (Rytel et al. 2013), it can be concluded that removing the potato skin in the process of peeling them, or, as in the production of juices, by separating the potato pulp during its production, from 50% to 70% of glycoalkaloids can be removed. The authors (Rytel et al. 2020; Tajner‐Czopek et al. 2008) also emphasize that further technological stages used in the production of fried or dried potato products contribute to the loss of glycoalkaloids to a lesser extent. Ready‐made potato products may still contain from a few to a dozen or so percent of the initial amount of these compounds that were in the raw material. Due to their highly toxic effects on living organisms, glycoalkaloids can have an adverse impact on human health, thereby limiting potato juice utilization (Zhang & Zhu, 2023). Considering the above, the determination of their content in juice obtained from new cultivars of colored‐flesh potatoes has been planned in the present study. However, based on the long‐lasting research of author's project (Panchal & Brown, 2022; Pęksa et al., 2013), it can be inferred that the contemporary potato cultivars intended for both processing and direct consumption have low levels of these compounds.

Another drawback is the natural tendency of potato juice to undergo enzymatic browning, which deteriorates the quality and sensory attractiveness of raw materials. Various solutions, including thermal processes, are employed to prevent undesirable color changes during processing. The high temperature applied to the raw material inactivates the enzymes involved in enzymatic browning processes (Lachman et al., 2008). Unfavorable color changes occurring in potato tubers result from enzymatic oxidation of polyphenols (including tyrosine or chlorogenic acids) by polyphenol oxidase and peroxidase (Lachman et al., 2005; Zhou et al., 2021). Pasteurization of potato juice can improve its color but may also modify the content and composition of anthocyanins. Therefore, the present study aimed to determine the impact of pasteurization at temperatures of 65°C and 75°C on the color and its stability, as well as the composition and content of anthocyanins in juice extracted from red‐fleshed and purple‐fleshed potatoes.

## 2 MATERIALS AND METHODS

### 2.1 Colored potato juices

The experimental material included juices extracted from three purple‐fleshed potato cultivars: Provita, Double Fun, and Violet Queen; and from three red‐fleshed potato cultivars: Magenta, Mulberry Beauty, and Lily Rose. The potatoes were sourced directly from Polish producers and were produced in the growing season 2020 and 2021.

#### 2.1.1 Getting juices

Juices were extracted from washed, dried, nonpeeled potatoes using an automatic juice extractor Robot Coupe J100, and left in a dark room for 45 min to enable starch separation via sedimentation. Afterward, they were filtered through a gauze and centrifuged using an MPW‐351R centrifuge at 1000 rpm and a temperature of 9°C for 10 min to achieve clear juices. The clear juices obtained from each cultivar were divided into three ca. 1.5‐L portions. The first portion was left nonpasteurized, whereas the two other portions were pasteurized at 65°C for 5 min and at 75°C for 5 min. After pasteurization, the juices were cooled and centrifuged on an MPW‐351R centrifuge at 3000 rpm, with a temperature of 9°C, for 5 min to obtain clear solutions. Approximately 1 L of nonpasteurized and pasteurized juices as well as ca. 1 kg of tubers of each analyzed cultivar were preserved by lyophilization for further analyses in a Christ Alpha 1–4 LSCplus apparatus (Osterode am Hatz, Germany) under the following parameters: pressure of 63 Pa, shelf heating temperature of 30°C, and time of 24 h. The resultant lyophilizes were stored at −18°C in closed containers.

### 2.2 Analytical methods

The raw material (potato tubers) (Table A1) as well as pasteurized and nonpasteurized juices (Table A2) were determined for the dry matter content (AOAC, 2002). In addition, the color of all juice samples was measured with the calorimetric method (Wrolstad et al., 2005). The lyophilized samples were analyzed in terms of: the content of glycoalkaloids (α‐chaconine and α‐solanine) (Nemś & Pęksa, 2018); the total polyphenol content (De Masi et al., 2020; Eichhorn & Winterhalter, 2005) and the content and composition of anthocyanins with the UHPLC MS/MS liquid chromatography method (Kaaber et al., 2002).

#### 2.2.1 Color with the Konica Minolta CR‐5 camera according to the Hunter scale (lab)

The color analysis of the juices was performed with a Konica Minolta CR‐200 measuring apparatus calibrated in Hunter scale L, a, and b units. Color measurements were conducted immediately after the preparation of the pasteurized and nonpasteurized juices as well as 1 and 4 h after their production (Muneta, 1981; Wrolstad et al., 2005).

Color space parameters, hue angle (h°) and chroma (C), were computed based on a * and b * values:

- Hue angle = Arctan (b*/a*)
- Chroma = ((a*^2^) + (b*^2^))^0.5^ (AOAC, 2002).

#### 2.2.2 Total glycoalkaloid content

Contents of α‐chaconine and α‐solanine were determined in 1 g of the lyophilized samples using a high‐pressure liquid chromatograph (HPLC, Shimadzu Prominence‐LC‐2030C Plus) equipped in an ultraviolet–visible detector, a Supelcosil LC column (18.25 cm × 4.6 mm, 5 μm), and a computer set for monitoring the HPLC. A mixture of acetonitrile and 0.1 M KH2PO4 (70:30 vol/vol) was used as the eluent. The process was run at a temperature of 70°C, a flow rate of 1 mL/min, and light with a wavelength of 200 nm. α‐solanine and α‐chaconine produced by Sigma‐Aldrich (Poznań, Poland) were used as standards. The glycoalkaloids (TGA), α‐solanine and α‐chaconine, were quantified by comparing their retention times and spectral characteristics of a diode matrix with those of respective standards (Rytel et al., 2013; Tajner‐Czopek et al., 2012).

#### 2.2.3 Extraction of polyphenols and anthocyanins

The lyophilized samples of potatoes and juices were extracted with a 70% aqueous acetone solution acidified with 0.1% acetic acid. Two‐gram samples of the lyophilized were collected for analysis. The mixture was mixed with a Vortex stirrer, then placed in a SONIC‐9 ultrasonic water bath for 5 min and centrifuged using an MPW‐351R centrifuge. The extraction was repeated two more times. Afterward, the acetone–water layer was separated using chloroform to remove lipophilic compounds. The color acetone–water fraction was collected and evaporated on a Büchi rotary evaporator (Merck, Darmstadt, Germany) until acetone had been completely removed. The remaining extract was brought to the volume of 5 mL using 50% methanol. The samples were stored in a freezer at −20°C until further analysis. Prior to chromatographic analyses, the samples were filtered through Nylon 6 0.22 μm filters (Pȩksa et al., 2002).

#### 2.2.4 Total polyphenolic content

The total polyphenolic content (TP) was determined with the Folin–Ciocalteu colorimetric method (Benzie & Strain, 1996). Determinations were performed on 0.1‐mL samples of extracts (prepared as in Section 2.2.3), completed with 2 mL of distilled water and 0.2 mL of the Folin–Ciocalteu reagent. Next, 1 mL of a 20% aqueous sodium carbonate solution was added to the mixture. After 1 h, absorbance was measured at the wavelength of 765 nm with the spectrophotometric method. The results were presented as milligrams of gallic acid (GAE/1 g of the sample) (Nemś, Pęksa, et al., 2015b; Yen & Chen, 1995).

#### 2.2.5 Quantification of anthocyanins by HPLC‐PDA

The content of anthocyanins was determined according to Kucharska (2012) using a Dionex (Waltham, MA, USA) HPLC system equipped with an Ultimate 3000 model of a diode array detector, an LPG‐3400A quaternary pump, an EWPS‐3000SI autosampler, and a TCC‐3000SD thermos‐stated column compartment, controlled by Chromeleon v.6.8. software. The Cadenza Imtakt column C5‐C18 (75 × 4.6 mm, 5 μm; Portland, USA) was used. The following solvents constituted the mobile phase: 4,5% formic acid (solvent A) and 100% acetonitrile (solvent B). The following elution conditions were applied: 0–1 min 5% B in A; 1–20 min 25% B in A; 20–27 min 100% B in A; 27–30 min 5% B in A. The flow rate was 1 mL/min, and the injection volume was 40 μL. The column was operated at 30°C. Anthocyanins were monitored at 520 nm and their content was expressed in cyanidin 3‐O‐glucoside equivalents (CygE)/100‐g dry mass (dm).

#### 2.2.6 Determination of the amount and composition of anthocyanins by liquid chromatography UHPLC MS/MS

The compounds were identified by means of the Acquity liquid chromatography system (UPLC) coupled with MS with quadrupole time‐of‐flight (Q‐TOF) (UPLC/Synapt Q‐TOF MS, Waters Corp., Milford, MA, USA), with ionization source provided by electrospraying (ESI). The separation was performed on an Acquity BEH C18 column (100 mm × 2.1 mm id, 1.7 μm; Waters), with a mixture (v/v) of 2.0% formic acid (A) and acetonitrile (B) as the mobile phase. The gradient program was as follows: initial conditions—1% B in A, 12 min 25% B in A, 12.5 min 100% B, 13.5 min 1% B in A. The flow rate was 0.45 mL/min and the sample injection volume was 5 μL. The column operated at a temperature of 30°C. Ultraviolet–visible absorption spectra were registered online during UPLC analysis, and spectral measurements were performed in the wavelength range of 200–600 nm, in 2 nm ramps. The main parameters of Q‐TOF MS work were as follows: capillary voltage: 2.0 kV; cone voltage: 40 V; gas flow rate on the cone: 11 L/h; collision energy: 28–30 eV; source temperature: 100°C; desolvation temperature: 250°C; collision gas: argon; desolvation gas (nitrogen) flow rate: 600 L/h; data acquisition range: *m*/*z* 100–2000 Da; ionization mode: negative and positive. Data were collected by means of Mass‐LynxTM V 4.1 software (Kucharska et al., 2017; Li et al., 2014. The content of anthocyanins was monitored at the wavelength of ƛ = 520 nm Kucharska, 2012).

#### 2.2.7 Statistical analysis

The results were subjected to one‐way and multi‐way analysis of variance using Statistica 13.1 package, where the least significant difference (LSD) and homogenous groups were determined with the Duncan's test at a significance level of α = .05.

Dry matter content, glycoalkaloid content as well as the content and composition of anthocyanins were determined in two laboratory replications, whereas determination of polyphenol content and color analysis were performed in six laboratory replications. The results presented in the manuscript represent the mean of the laboratory replications and two study years.

## 3 RESULTS AND DISCUSSION

### 3.1 Characteristics of nonpasteurized potato juices

Novel raw materials introduced to the market for consumption or food processing should be monitored for the presence of toxic compounds. Natural toxic substances found in potatoes are glycoalkaloids (TGA). Their adverse impact on the human body has been extensively described in the literature, and their toxicity is comparable to that of strychnine or arsenic (Rytel et al., 2019). In most European countries, including Poland, the permissible content of total glycoalkaloids (TGA) in potatoes is 20 mg TGA/100 g tuber fresh matter (f.m.). In potatoes, α‐chaconine and α‐solanine contents account for 95% of the total glycoalkaloid content, whereas the remaining 5% are represented by β_1_‐, β_2_‐, and γ‐ solanine as well as β_1_‐, β_2_‐, and γ‐chaconine. In most potato cultivars, the content of α‐chaconine is higher than that of α‐solanine (Barceloux, 2008). The analyzed colored‐fleshed potato cultivars differed in the contents of glycoalkaloids (Table 1). The α‐solanine to α‐chaconine ratio ranged from 1:2.3 (in tubers of the red‐fleshed cultivar Magenta Love) to 1:4.3 (in tubers of the purple‐fleshed cultivar Violet Queen). In our previous study (Rytel et al., 2013), this ratio was found to range from 1:1.8 to 1:2.1 for colored‐fleshed potato cultivars. Similar results were reported in the research by Tajner‐Czopek et al. (2012), where the α‐solanine‐to‐α‐chaconine ratio ranged from 1:1.8 to 1:2.2. The mean total content of glycoalkaloids reached 4.88 TGA/100 g f.m. in colored‐fleshed potato tubers, 4.78 mg TGA/100 g f.m. in purple‐fleshed potato tubers, and 5.0 mg TGA/100 g f.m. in red‐fleshed cultivars (Table 1). As shown in our previous study (Rytel et al., 2013), the TGA reached 5.68 mg TGA/100 g f.m. in purple‐fleshed potato cultivars and 5.26 mg TGA/100 g f.m. in the red‐fleshed ones. In turn, the mean TGA content reported by Kita et al. (2023) for purple‐fleshed potato tubers of Provita cultivar reached 4.11 mg TGA/100 g f.m.

**TABLE 1:** The total glycoalkaloid (TGA) content in potatoes (mg/100 f.m.) with purple or red flesh (average of 2 years).

Potato variety	α‐Solanine	α‐Chaconine	TGA
Provita	1.96^e^	6.46^e^	8.42^f^
Double Fun	0.93^c^	2.98^b^	3.92^c^
Violet Queen	0.37^a^	1.62^a^	1.99^a^
Magenta Love	1.55^d^	3.61^c^	5.17^d^
Mulberry Beauty	2.03^e^	5.13^d^	7.16^e^
Lily Rose	0.54^b^	2.11^a^	2.65^b^
LSD	0.16	.60	0.63

*Note*: Data are expressed as the mean, *n* = 6. Results in the same column with the same superscript were not statistically significant (*p* < .05) according to the classification obtained by Duncan's test as determined by one‐way ANOVA.

Abbreviation: LSD, last significant difference.

In the present study, also juices were made of the analyzed potato cultivars. In the juices extracted from the purple‐fleshed cultivars, the glycoalkaloid content (Table 2) ranged from 1.08 mg TGA/100 g potato juice (Violet Queen cultivar) to 3.72 mg TGA/100 g f.m. (Provita cultivar). In turn, the juices made of the red‐fleshed potato tubers contained 1.59 mg TGA/100 g f.m. (Lily Rose cultivar) to 2.88 mg TGA/100 g f.m. (Mulberry Beauty cultivar) (Table 1). Kowalczewski et al. (2021) demonstrated a significant effect of light‐colored (yellow‐fleshed) potato cultivars on the content of glycoalkaloids in juices extracted from their tubers. The juices made of tubers having a higher TGA content also had a higher content of these compounds. The nonpasteurized juices extracted from the colored‐fleshed potato tubers had a 2‐fold lower glycoalkaloid content (mean: 2.13 mg TGA/100 g potato juice) compared to potato tubers (mean: 4.9 mg TGA/100 g f.m.) (Table 1). The lower TGA content found in juices may be due to the removal of part of precipitate formed during their preparation. Kowalczewski et al. (2016) demonstrated that part of the toxic compounds diffused to potato juice during extraction.

**TABLE 2:** Glycoalkaloid content (mg/100 f.m.) in juices from potatoes with red and purple flesh (average of 2 years).

Variant	Variety of potato juice	α‐Solanine	α‐Chaconine	TGA
Nonpasteurized juice	Provita	0.84^f^	2.88^c^	3.72^d^
65°C	Provita	0.66^d^	1.90^c^	2.56^c^
75°C	Provita	0.43^c^	1.20^bc^	1.63^b^
Nonpasteurized juice	Double Fun	0.26^b^	1.13^bc^	1.40^ab^
65°C	Double Fun	0.16^ab^	0.49^ab^	0.64^a^
75°C	Double Fun	0.17^ab^	0.50^ab^	0.68^a^
Nonpasteurized juice	Violet Queen	0.26^b^	0.82^b^	1.08^ab^
65°C	Violet Queen	0.09^a^	0.27^a^	0.36^a^
75°C	Violet Queen	0.04^a^	0.20^a^	0.24^a^
Nonpasteurized juice	Mulberry Beauty	0.70^e^	2.18^bc^	2.88^bc^
65°C	Mulberry Beauty	0.44^c^	0.77^b^	1.21^ab^
75°C	Mulberry Beauty	0.39^bc^	0.86^b^	1.25^h^
Nonpasteurized juice	Magenta Love	0.65^d^	1.47^c^	2.12^bc^
65°C	Magenta Love	0.36^bc^	0.54^ab^	0.90^a^
75°C	Magenta Love	0.39^bc^	0.63^ab^	1.02^a^
Nonpasteurized juice	Lily Rose	0.32^bc^	1.27^bc^	1.59^ab^
65°C	Lily Rose	0.23^b^	0.59^ab^	0.82^a^
75°C	Lily Rose	0.10^a^	0.48^ab^	0.59^a^
	LSD	0.13	0.50	0.60

*Note*: Data are expressed as the mean, *n* = 6. Results in the same column followed by different letters indicate significant differences according to Duncan's test at *p* < .05 between variant and variety of potato juices as determined by two‐way ANOVA.

Abbreviation: LSD, last significant difference.

Likewise, the study conducted by Kowalczewski et al. (2021) showed no effect of potato juice production method on the α‐solanine‐to‐α‐chaconine ratio in the juices. In the analyzed nonpasteurized juices, this ratio ranged from 1:2.2 (juices from red‐fleshed Magenta Love cultivar) to 1:4.3 (juices from purple‐fleshed Double Fun cultivar) (Table 2). As reported by Kowalczewski, Olejnik, Białas, Kubiak, et al. (2019c), the α‐solanine‐to‐α‐chaconine ratio reached 1:0.04 in juices made of potato tubers with traditional light flesh. On the other hand, glycoalkaloids found in small contents in potato juices (below 10 mg/100 g f.m.) may confer health benefits to the human body as they exhibit strong antioxidative and anticarcinogenic properties (Barceloux, 2008; Friedman, 2006).

Potato juice contains also other biologically active substances with strong antioxidative properties, like, for example, polyphenolic compounds. The total polyphenol content determined in nonpasteurized juices extracted from the analyzed red‐fleshed and purple‐fleshed potato cultivars ranged from 285 mg GAE/100 g d.m. (juice from potatoes of Lily Rose cultivar) to 683 mg GAE/100 g d.m. (juice from potatoes of Violet Queen cultivar) (Table 3). Its higher value (by 6%) was determined in the juices made of the purple‐fleshed cultivars compared to those produced from the red‐fleshed potato tubers. The highest total polyphenol content was assayed in the juice produced from potatoes of Violet Queen cultivar (Table 3). Rytel et al. (2019) also demonstrated a higher total polyphenol content in the juices made of the purple‐fleshed potatoes. According to our previous study (Kowalczewski et al., 2012), the mean total content of polyphenols in the juices produced from colored‐fleshed potatoes reached 6.36 mgGAE/1 g (636 mgGAE/100 g d.m.). The total polyphenolic content determined in the juices extracted from the colored‐fleshed potato cultivars investigated in the present study was similar to that reported elsewhere (Rytel et al., 2013; Tajner‐Czopek et al., 2012). According to Kowalczewski et al. (2021); Kowalczewski, Olejnik, Białas, Rybicka, et al. (2019a), the juices produced from potato with light flesh color (yellow or cream) contained 5.82–12.85 mg GAE/1 g d.m. (582–1285 mg GAE/100 g d.m.) of phenolic compounds.

**TABLE 3:** Content of total polyphenols (mgGAE/100 g dm) and anthocyanins (mg/100 g dm) in potato juices of red and purple flesh varieties (average of 2 years).

Variant	Variety of potato juice	Total polyphenols	Anthocyanins
Nonpasteurized juice	Provita	299^b^	28.2^ł^
65°C	Provita	394^i^	9.57^c^
75°C	Provita	370^f^	12.2^f^
Nonpasteurized juice	Double Fun	611^L^	80.2°
65°C	Double Fun	962^n^	41.8^m^
75°C	Double Fun	983°	6.39^a^
Nonpasteurized juice	Violet Queen	683^m^	134.2^r^
65°C	Violet Queen	664^ł^	94.4^p^
75°C	Violet Queen	564^k^	61.0^n^
Nonpasteurized juice	Mulberry Beauty	379^g^	25.8^L^
65°C	Mulberry Beauty	352^e^	22.1^i^
75°C	Mulberry Beauty	384^h^	25.7^k^
Nonpasteurized juice	Magenta Love	467^j^	25.4^j^
65°C	Magenta Love	325^d^	9.33^b^
75°C	Magenta Love	377^g^	9.85^d^
Nonpasteurized juice	Lily Rose	285^a^	14.1^g^
65°C	Lily Rose	282^a^	15.1^h^
75°C	Lily Rose	307^c^	10.0^e^
	LSD	4.80	0.08

Data are expressed as the mean, *n* = 12, *n* = 6. Results in the same column followed by different letters indicate significant differences according to Duncan's test at *p* < .05 between variant and variety of potato juices as determined by two‐way ANOVA.

Abbreviation: LSD, last significant difference.

The polyphenolic compounds found in potatoes include, i.a., anthocyanins. Pelargonidin derivatives were identified in the juices made of the red‐fleshed potato cultivars (Table 5), whereas petunidins, malvidins, peonidins, delfhinidins, and cyanidins (with Petunidin and peonidin found to be the major identified anthocyanin) were identified in those made of the purple‐fleshed tubers (Table 4). According to Eichhorn and Winterhalter (2005), petunidin is the major anthocyanin of purple‐fleshed potatoes, whereas pelargonidin—of the red‐fleshed ones. Other authors (Backleh et al., 2004) have also identified petunidin, malvidin, and peonidin in purple‐fleshed potato tubers. Anthocyanins differ in structure but usually occur in the form of glycosides, that is, complexes with monosaccharides at positions 3 and 5 (most of the structural variations due to glycosidic substitution at the 3 and 5 positions and possible acylation of sugar residues with organic acids). The type of anthocyanins present in plants, their complexes with metals, and the presence of other pigments (e.g., carotenoids) affect their color (Nemś, Miedzianka, et al., 2015a).

**TABLE 4:** Anthocyanins identified in juices from red‐fleshed potatoes.

Variety	Peak	*t*_*r*_ (min)	[M]^+^ (*m/z*)	MS/MS (*m/z*)	Compound
Mulberry Beauty	1	4.97	741.1634	579/433/271	Pelargonidin 3‐rutinoside 5‐glucoside
	2	6.35	579.1962	271	Pelargonidin derivative
	3	7.23	887.0988	725/433/271	Pelargonidin 3‐coumaroylrutinoside 5‐glucoside izomer 1
	4	7.42	903.0843	741/433/271	Pelargonidin 3‐caffeoylrutinoside 5‐glucoside
	5	7.71	887.0988	725/433/271	Pelargonidin 3‐coumaroylrutinoside 5‐glucoside izomer 2
	6	8.12	887.1049	725/433/271	Pelargonidin 3‐coumaroylrutinoside 5‐glucoside izomer 3
	7	8.38	917.0928	755/433/271	Pelargonidin 3‐feruloylrutinoside 5‐glucoside
	8	9.00	887.0927	725/433/271	Pelargonidin 3‐coumaroylrutinoside 5‐glucoside izomer 4
	9	9.10	887.0763	725/433/271	Pelargonidin 3‐coumaroylrutinoside 5‐glucoside isomer 5
Magenta Love	1	4.98	741.1634	579/433/271	Pelargonidin 3‐rutinoside 5‐glucoside
	2	6.37	887.1049	725/474/271	Pelargonidin derivative
	3	6.53	887.1049	725/433/271	Pelargonidin 3‐coumaroylrutinoside 5‐glucoside isomer 1
	4	7.11	903.0905	287	Cyanidin derivative
	5	7.27	903.0905	725/433/271	Pelargonidin 3‐coumaroylrutinoside 5‐glucoside isomer 2
	6	7.41	903.0905	741/433/271	Pelargonidin 3‐caffeoylrutinoside 5‐glucoside
	7	7.72	887.1049	725/433/271	Pelargonidin 3‐coumaroylrutinoside 5‐glucoside isomer 3
	8	8.11	887.1049	725/433/271	Pelargonidin 3‐coumaroylrutinoside 5‐glucoside isomer 4
	9	8.39	917.0989	755/433/271	Pelargonidin 3‐feruloylrutinoside 5‐glucoside
	10	8.70	903.0905	287	Cyanidin derivative
	11	9.09	887.1049	725/433/271	Pelargonidin 3‐coumaroylrutinoside 5‐glucoside isomer 5
Lily Rose	1	4.97	741.1578	579/433/271	Pelargonidin 3‐rutinoside 5‐glucoside
	2	6.34	474.3375	271	Pelargonidin derivative
	3	7.23	887.0927	725/433/271	Pelargonidin 3‐coumaroylrutinoside 5‐glucoside izomer 1
	4	7.41	903.0844	741/433/271	Pelargonidin 3‐caffeoylrutinoside 5‐glucoside
	5	7.71	887.1049	725/433/271	Pelargonidin 3‐coumaroylrutinoside 5‐glucoside isomer 2
	6	8.11	887.1049	725/433/271	Pelargonidin 3‐coumaroylrutinoside 5‐glucoside isomer 3
	7	8.38	917.0865	755/433/271	Pelargonidin 3‐feruloylrutinoside 5‐glucoside
	8	8.70	887.0988	725/433/271	Pelargonidin 3‐coumaroylrutinoside 5‐glucoside izomer 3
	9	9.09	887.0988	725/433/271	Pelargonidin 3‐coumaroylrutinoside 5‐glucoside isomer 4

### 3.2 Color of nonpasteurized juices

**FIGURE 1:**
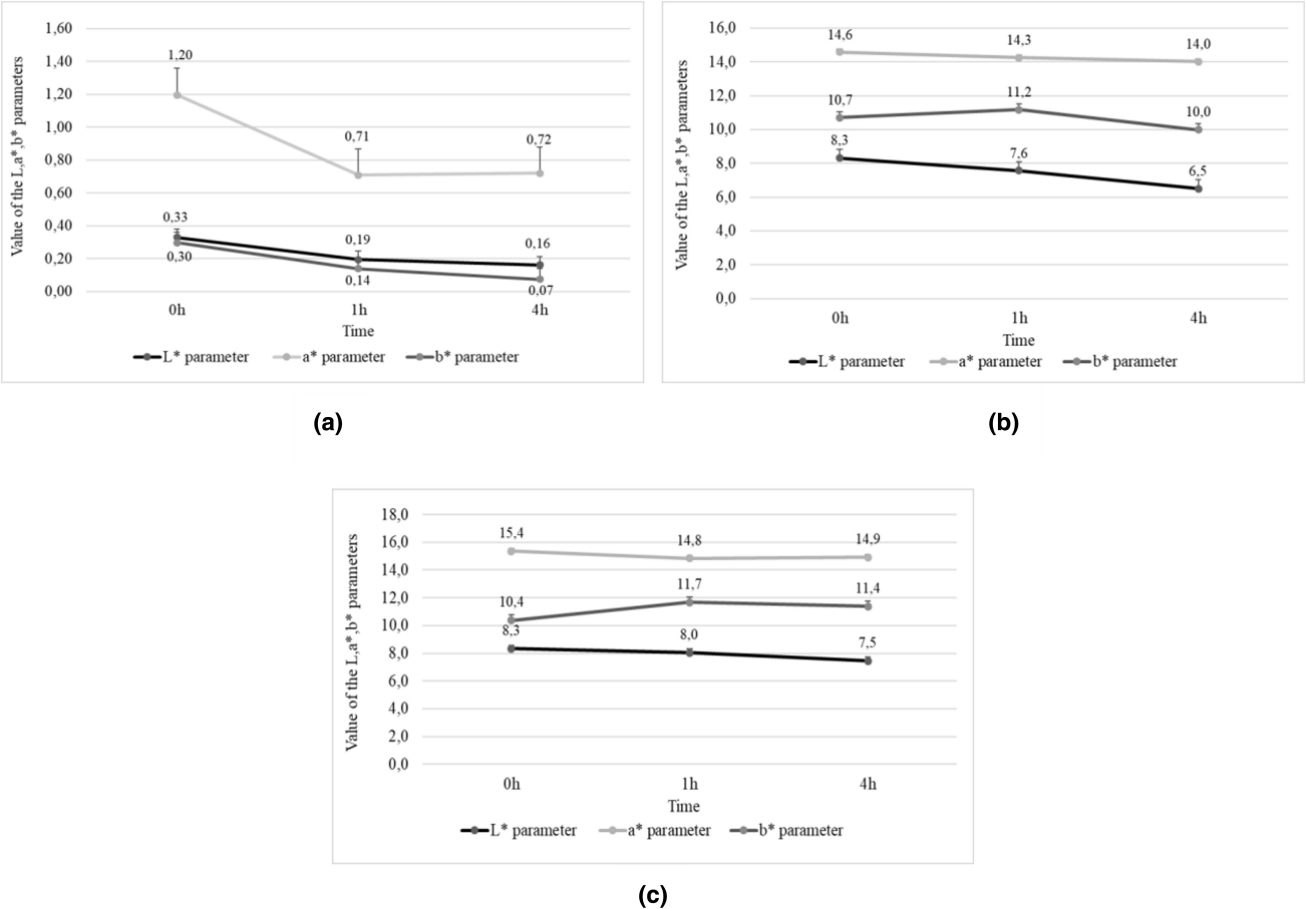
Value of the L*, a*, b* parameters from potato juices of purple flesh varieties (a) non‐pasteurized juices, (b) juices pasteurized at 65°C for 5 min, (c) juices pasteurized at 75°C for 5 min.

**FIGURE 2:**
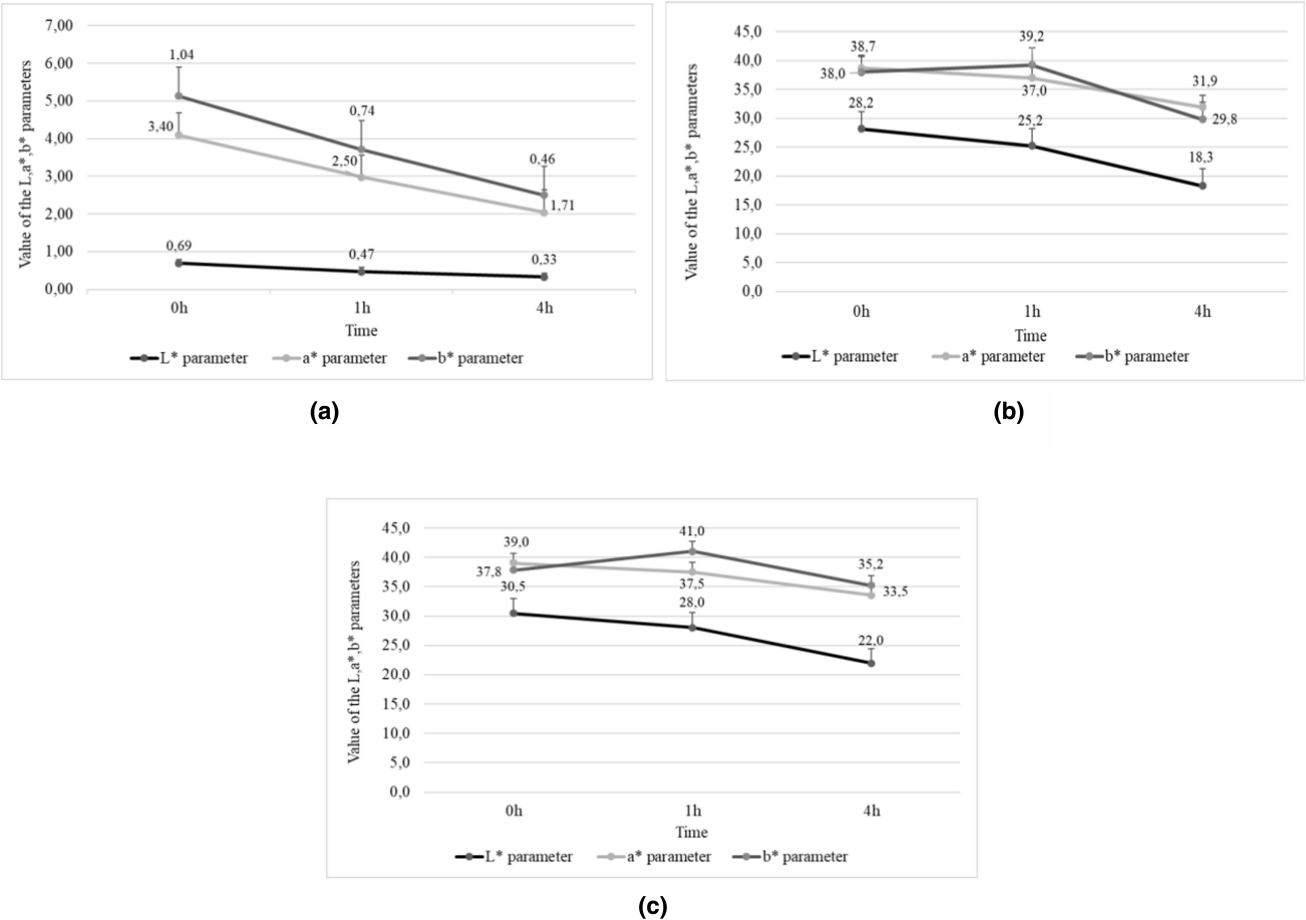
Value of the L*, a*, b* parameters from potato juices of red flesh varieties: (a) non‐pasteurized juices, (b) juices pasteurized to 65°C for 5 min, (c) juices pasteurized 75°C for 5 min.

When analyzed immediately after extraction, the potato nonpasteurized juice produced from purple‐fleshed tubers was darker (mean L* = 0.33) (Figure 1) compared to the juice made of the red‐fleshed tubers (mean L* = 0.69), (Figure 2) which was consistent with the findings reported by Iborra‐Bernad et al. (2014) and Rytel et al. (2019). The juice extracted from purple‐fleshed potatoes had a less contribution of red color (mean a* = 3.4) (Figure 1) than that made of the red‐fleshed cultivars (mean a* = 1.2) (Figure 2). In addition, immediately after extraction, the color of juices made of the purple‐fleshed potato tubers had a slight contribution of blue hue (mean b* = 0.30), (Figure 1) whereas yellow hue prevailed among the juices made of the red‐fleshed tubers (mean b* = 1.04) (Figure 2). Similar results were reported by other authors (Backleh et al., 2004) during color analysis of the colored‐fleshed potato cultivars.

In the nonpasteurized juice, the L* value decreased with time (Figures 1, 2). The mean value of the L* parameter determined for the juices made of the purple‐fleshed tubers ranged from 0.33 (immediately after extraction) to 0.16 (4 h after extraction). In turn, the lightness of juices produced from red‐fleshed potato cultivars ranged from 0.69 to 0.33 (Figure 2). With time, the color of purple‐fleshed potato juices showed a greater contribution of green; the a* value increased to 1.20 after 1 h since extraction and then dropped to 0.72 after 4 h (Figure 1). In turn, the value of a* parameter decreased from 1.20 to 0.72, indicating a color shift toward a greater contribution of green (Table A3, Figure 1). In the case of the red‐fleshed potato cultivars (Table A3, Figure 2), the value of a* parameters decreased from 3.4 (after 1 h) to 1.71 (after 4 h since juice extraction). In turn, the value of b* parameter in purple juices decreased from 0.30 to 0.07 (Figure 1), indicating a color shift towards a lower contribution of yellow. However, the change in the b* value parameter in red juices (from 1.04 to 0.46) (Figure 2) indicated a color shift towards blue. Rytel et al. (2019) demonstrated similar correlations while analyzing the color of potato tubers and its changes over time.

**FIGURE 3:**
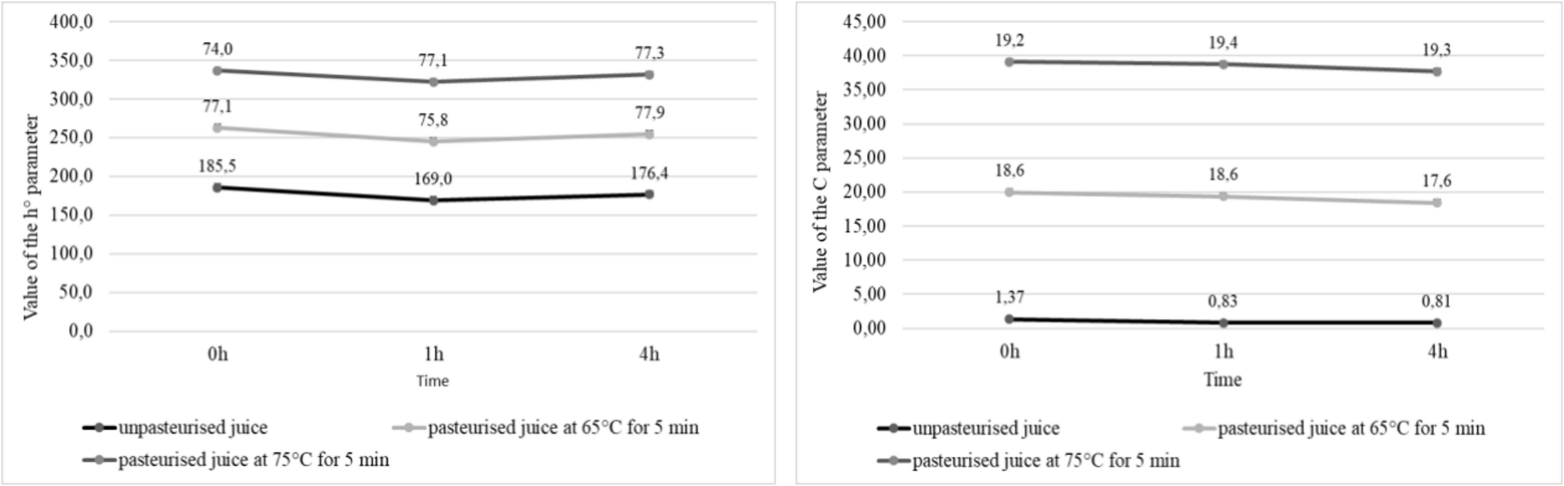
Value of the h° and C parameters from potato juices of purple flesh varieties.

**FIGURE 4:**
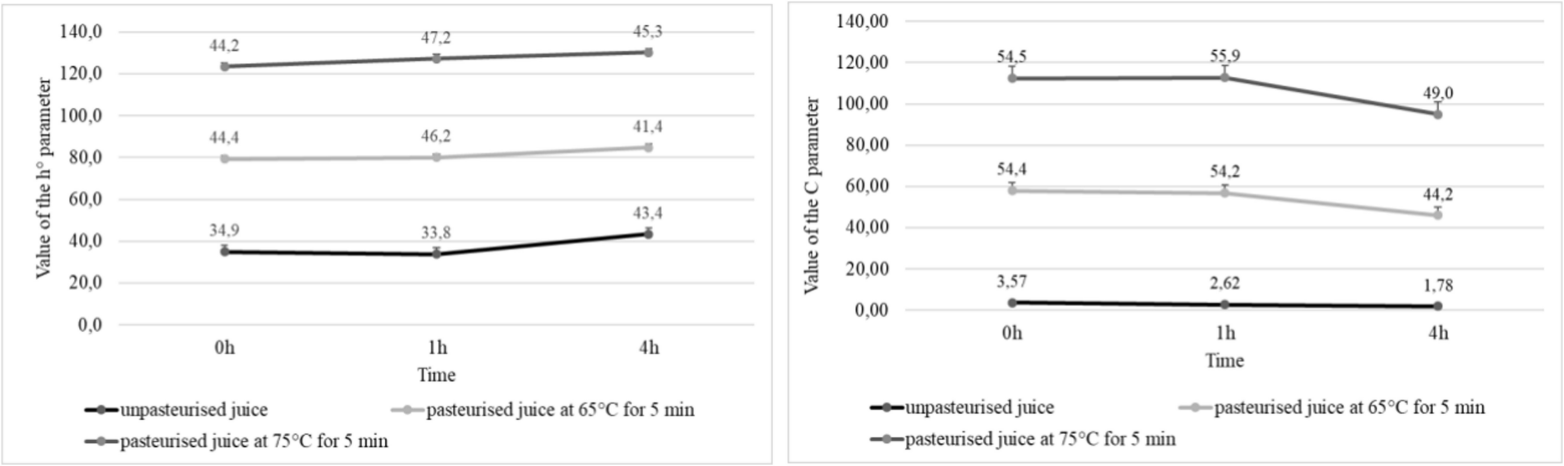
Value of the h° and C parameters from potato juices of red flesh varieties.

**FIGURE 5:**
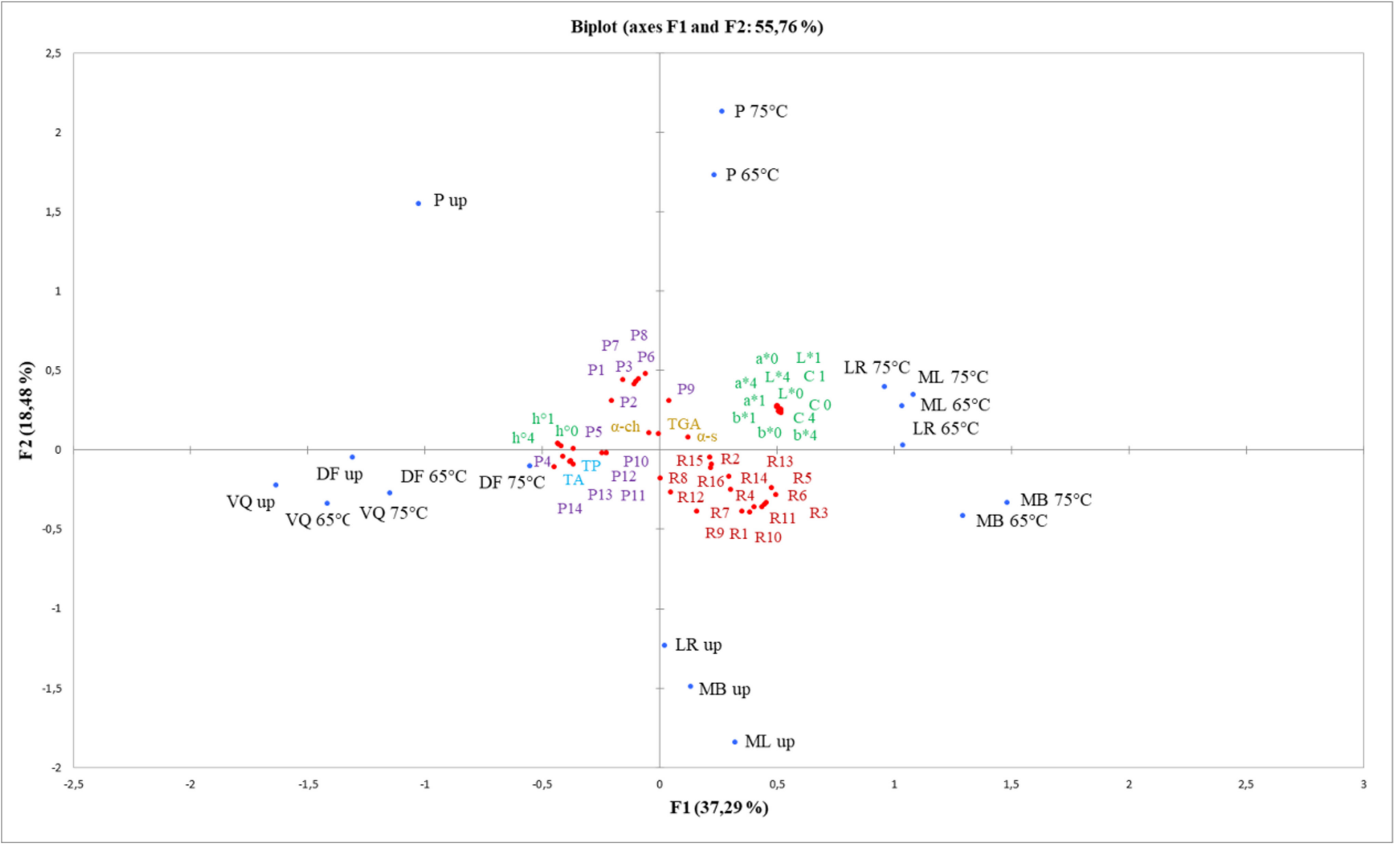
PCA all chemical components and properties related to juices of potato variety and temperature of pasteurization.

The calculated values of h° parameter indicated a juice color shift over time in the case of both purple‐fleshed and red‐fleshed cultivars. The mean value of hue angle (parameter h°) of the juices made of purple‐fleshed potatoes decreased from 185.5 immediately after extraction to 176.4 after 4 h since extraction (Figure 3). In the case of the juice produced from red‐fleshed cultivars, the h° values increased from 34.9 to 43.4, respectively (Figure 4). Decreases were also noted in the chromaticity of potato juices (parameter C). In the juices made from purple‐fleshed cultivars, its value decreased from 1.37 immediately after extraction to 0.81 after 4 h since extraction (Figure 3), whereas in the juices produced from red‐fleshed tubers the respective values were from 3.57 to 1.78 (Figure 4). Juices extracted from other vegetables, for example, sweet potatoes, showed significantly higher values of color parameters. According to Rios‐Romero et al. (2021), the juice from orange sweet potatoes had a chroma value at C = 41.0 and hue angle at h° = 58.1 (Figure 5).

### 3.3 Effect of pasteurization temperature on the color and contents of glycoalkaloids and biologically active compounds of potato juices

In spite of that, the pasteurization of juices at 65°C and 75°C for 5 min contributed to TGA decrease (Table 2).

The TGA content was, on average, 1.08 TGA/100 g f.m. in the juices pasteurized at 65°C for 5 min and 0.9 TGA/100 g f.m. in the juices pasteurized at 75°C for 5 min. After pasteurization, the content of α‐chaconine decreased, on average, by 0.91 mg TGA/100 g juice and that of α‐solanine by 0.22 mg TGA/100 g juice, compared to the nonpasteurized juices (Table 2). The greater losses of α‐chaconine may be due to its better solubility and its greater tendency for degradation compared to α‐solanine, both of which result from differences in the chemical structure of these compounds. A sugar component of α‐solanine is solatriose, composed of glucose, galactose, and rhamnose bound with solanidine by galactose. In turn, α‐chaconine is constituted by two molecules of rhamnose and glucose and is bound with solanidine (Kowalczewski et al., 2012; Kowalczewski, Olejnik, Białas, Rybicka, et al., 2019a; Rytel et al., 2020). Rytel (2012) found such a dependency during potato blanching, whereas an opposite tendency during potato steam cooking, which resulted in greater α‐solanine losses that were probably due to the water vapor effect and a lack of a washing out agent. Tajner‐Czopek et al. (2008) observed smaller losses of α‐solanine than α‐chaconine during technological processing. Another factor affecting the usability of juices for food processing or direct consumption is the α‐solanine‐to‐α‐chaconine ratio in tubers, due to higher toxicity of α‐chaconine (Kowalczewski et al., 2016, 2021). This ratio was lower in the pasteurized juices and ranged from 1:1.5 (red‐fleshed Magenta Love cultivar) to 1:5 (purple‐fleshed Violet Queen cultivar). The mean α‐chaconine content in the juices pasteurized at the lower tested temperature reached 0.76 mg/100 g f.m. and that of = α‐solanine reached 0.32 mg/100 g f.m., whereas the respective values determined in the juices pasteurized at the higher temperature tested were at 0.65 mg/100 g f.m. and 0.25 mg/100 g f.m. (Table 2).

As Pęksa et al. (2016) claim, the initial stages of juice preparation for coagulation (protein separation), including washing, drying, and starch and potato fiber separation, may contribute to glycoalkaloid content reduction in the juice. Many authors (Felczak, 2021; Rytel, 2010) have found the highest TGA content in the potato skin, directly underneath it; therefore, the pretreatment of raw tubers has been reported to contribute to even 50% losses of glycoalkaloids (Kowalczewski et al., 2012, 2016, 2021; Kowalczewski, Olejnik, Białas, Rybicka, et al., 2019a). In addition, as Kita et al. (2023) claim, purple‐fleshed potato tubers of Provita cultivar contain approximately 40% TGA less compared to potato cultivars with traditional yellow‐colored flesh. This potentially increases the applicability of juices extracted from colored‐fleshed cultivars.

Pasteurization temperature was found to affect the contents of total polyphenols and anthocyanins in juices extracted from the analyzed potato cultivars (Table 3). The extent of changes in total polyphenol content of the juices varied and depended also on the potato cultivar. Pasteurized juices made from red‐fleshed potatoes on average 10% less total compared to nonpasteurized juices (Table 3). However, no losses of polyphenols were noted in the juices extracted from the purple‐fleshed cultivars Double Fun and Provita and red‐fleshed cultivar Lily Rose (Table 3). This lack of losses may be due to the chemical structure of polyphenolic compounds and the cascade of reactions occurring in the samples during high‐temperature treatment (Iborra‐Bernad et al., 2014; Kowalczewski, Olejnik, Białas, Kubiak, et al., 2019c; Rios‐Romero et al., 2021). The diversified chemical structure causes the antioxidants to convert from glycosidic forms to more active aglycone. In addition, it contributes to the formation of new pro‐oxidants and antioxidants as well as complexes with other food constituents (Nazarenko et al., 2002). Scientific literature lacks works that would corroborate the effect of pasteurization on either increasing or decreasing total polyphenol content in food. It may, however, be posited that the impact of heat‐treatment processes on the contents of biologically active compounds is determined by such factors as: type of food product or raw material; type, temperature, and duration of heat treatment; as well as losses of antioxidant vitamins during this treatment (Danesi & Bordoni, 2008; Dewanto et al., 2002; Ismail et al., 2004; McDougall et al., 2010; Różańska et al., 2014; Ševčík et al., 2009; Tudela et al., 2002; Turkmen et al., 2005; Wachtel‐Galor et al., 2008). In the food industry, the pasteurization process applies most often to hot‐pressed juices. The potato juices analyzed in the present study were cold‐pressed to minimize losses of biologically active compounds. This assumption has been confirmed by Genova et al. (2016), who observed an increase in the content of biologically active compounds in cold‐pressed juices compared to the hot‐pressed ones. These authors hypothesized that the results achieved might have been due to a temporary temperature increase promoting the release of phenolic compounds bound with fragments of cell walls in juices.

The mean total polyphenol content determined in the juices made of the purple‐fleshed cultivars and pasteurized at 65°C reached 673 mg GAE/100 g d.m., whereas in those pasteurized at 75°C it was 639 GAE/100 g d.m. In turn, the mean contents of these compounds in the juices extracted from red‐fleshed potato cultivars and pasteurized at 65°C and 75°C reached 320 and 356 mg GAE/100 g d.m., respectively. In the study conducted by LSP Basílio et al. (2022), the content of phenolic compounds in potato juice pressed from purple‐fleshed tubers pasteurized at 80°C for 3 min reached 0.7 mg GAE/1 g d.m. (70 mg GAE/100 g d.m.). In contrast, Rios‐Romero et al. (2021) did not show any significant changes in the total polyphenolic content of the juice made from the purple‐fleshed potato cultivar compared to the juice steam‐treated for 2 min. Furthermore, Nemś, Pęksa, et al. (2015b) reported 3.27 mg GAE/1 g d.m. (327 mg GAE/100 g d.m.) of total polyphenols in red‐fleshed and purple‐fleshed potato tubers, whereas their later study (Nemś & Pęksa, 2018) demonstrated the mean total polyphenolic content of 0.15 mg GAE/1 g d.m. (15 mg GAE/100 g d.m.) in fried snacks made of the same potato cultivars.

The temperatures used in the juice pasteurization process also influenced the composition and anthocyanin content. Pasteurized juices contained on average approximately 30% less anthocyanins compared to unpasteurized juices. Anthocyanin losses in the tested juices depended largely on the potato variety used for their production. Juices from red‐colored potatoes contained mainly pelargonidin and its derivatives, which turned out to be more thermostable than anthocyanins determined in juices from purple‐fleshed potatoes. Purple juices contained the most petunidin and peonidin and their derivatives, but after pasteurization, the largest share was petunidin 3‐feruloylrutinoside 5‐glucoside (Tables 6, 7).

**TABLE 5:** Anthocyanins identified in juices from purple‐fleshed potatoes.

Variety	Peak	*t*_*r*_ (min)	[M]^+^ (*m/z*)	MS/MS (*m/z*)	Compound
Provita	1	4.59	787.137	625/479/317	Petunidin 3‐rutinoside 5‐glucoside
	2	5.17	771.1607	609/463/301	Peonidin 3‐rutinoside 5‐glucoside
	3	7.04	949.0657	787/479/317	Petunidin 3‐caffeoylrutinoside 5‐glucoside
	4	7.18	919.0762	757/465/303	Delphinidin 3‐coumaroylrutinoside 5‐glucoside
	5	7.55	933.0889	771/463/301	Peonidin derivative
	6	7.66	933.0889	771/479/317	Petunidin 3‐coumaroylrutinoside 5‐glucoside
	7	7.95	695.3319	479/317	Petunidin 3‐feruloylrutinoside 5‐glucoside
	8	8.23	917.0989	755/463/301	Peonidin 3‐coumaroylrutinoside 5‐glucoside
	9	7.98	947.0879	785/463/301	Peonidin 3‐feruloylrutinoside 5‐glucoside
Double Fun	1	5.19	771.1494	609/463/301	Peonidin 3‐ rutinoside 5‐glucoside
	2	6.50	474.337	331	Malvidin derivative
	3	7.05	949.0657	787/479/317	Petunidin 3‐caffeoylrutinoside 5‐glucoside
	4	7.20	919.0699	303	Unidentified
	5	7.51	963.0695	801/493/331	Malvidin derivative
	6	7.69	933.0763	771/479/317	Petunidin 3‐coumaroylrutinoside 5‐glucoside
	7	7.86	963.0630	801/479/317	Petunidin 3‐feruloylrutinoside 5‐glucoside
	8	8.20	947.0879	785/493/331	Malvidin 3‐coumarylorutinoside 5‐glucoside isomer 1
	9	8.48	977.0734	815/493/331	Malvidin 3‐feruloylrutinoside 5‐glucoside
	10	8.70	947.0879	785/493/331	Malvidin 3‐coumarylorutinoside 5‐glucoside isomer 2
	11	9.06	917.0989	755/463/301	Peonidin 3‐coumarylorutinoside 5‐glucoside
Violet Queen	1	4.60	787.137	625/479/317	Petunidin 3‐ rutinoside 5‐glucoside
	2	5.29	801.145	639/493/331	Malvidin 3‐ rutinoside 5‐glucoside
	3	6.01			Unidentified
	4	6.68			Unidentified
	5	7.03	949.0657	787/479/317	Petunidin 3‐caffeoylrutinoside 5‐glucoside
	6	7.16	949.0657	771/479/317	Petunidin 3‐coumaroylrutinoside 5‐glucoside isomer 1
	7	7.55	933.0763	771/479/317	Petunidin 3‐coumaroylrutinoside 5‐glucoside isomer 2
	8	7.66	933.0763	771/479/317	Petunidin 3‐coumaroylrutinoside 5‐glucoside isomer 3
	9	8.01	963.0694	801/479/317	Petunidin 3‐feruloylrutinoside 5‐glucoside
	10	8.24	947.0879	785/493/331	Malvidin 3‐coumaroylrutinoside 5‐glucoside
	11	8.48	977.0734	815/493/331	Malvidin 3‐feruloylrutinoside 5‐glucoside
	12	8.78	933.0763	771/479/317	Petunidin 3‐coumaroylrutinoside 5‐glucoside isomer 4

**TABLE 6:** Content of identified anthocyanins (mg/100 g dm) in pasteurized and unpasteurized purple‐fleshed potato varieties (average of 2 years).

Compound	Provita			Double fun			Violet queen		
	Nonpasteurized	65°C	75°C	Nonpasteurized	65°C	75°C	Nonpasteurized	65°C	75°C
Petunidin 3‐rutinoside 5‐glucoside	0.72	0.39	0.53	—	—	—	0.31	—	—
Peonidin 3‐rutinoside 5‐glucoside	0.39	0.28	0.27	0.64	—	—	—	—	—
Delphinidin 3‐coumaroylrutinoside 5‐glucoside	0.83	0.35	0.44	—	—	—	—	—	—
Petunidin 3‐coumaroylrutinoside 5‐glucoside	8.61	1.96	2.38	12.8	4.61	—	97.0	62.0	40.6
Petunidin 3‐feruloylrutinoside 5‐glucoside	0.64	0.45	0.49	—	—	—	4.81	3.75	1.00
Peonidin 3‐coumaroylrutinoside 5‐glucoside	15.7	5.38	7.25	—	—	—	—	—	—
Peonidin 3‐feruloylrutinoside 5‐glucoside	0.89	0.48	0.50	—	—	—	—	—	—
Unidentified	0.35	0.28	0.32	—	—	—	—	—	—
Malvidin derivative	—	—	—	0.84	0.58	—	—	—	—
Malvidin 3‐coumarylorutinoside 5‐glucoside isomer 1	—	—	—	60.9	33.0	5.97			—
Malvidin 3‐feruloylrutinoside 5‐glucoside	—	—	—	4.56	2.81	—	1.34	1.04	0.84
Malvidin 3‐coumarylorutinoside 5‐glucoside isomer 2	—	—	—	0.55	0.77	0.42	—	—	—
Petunidin 3‐caffeoylrutinoside 5‐glucoside	—	—	—	—	—	—	3.93	6.37	1.69
Malvidin 3‐coumaroylrutinoside 5‐glucoside	—	—	—	—	—	—	26.9	21.2	16.8

Other authors (Rytel et al., 2021) also found that processes using higher temperatures contribute to large losses of anthocyanins. The authors also found different thermostability of anthocyanins, indicating that pelargonidin‐3‐feruloylrutinoside‐5‐glucoside and pelargonidin‐3‐caffeoylrutinoside‐5‐glucoside are more stable (Table 7).

**TABLE 7:** Content of identified anthocyanins (mg/100 dm) in pasteurized and unpasteurized red‐fleshed potato varieties (average of 2 years).

Compound	Mulberry beauty			Magenta love			Lily rose		
	Nonpasteurized	65°C	75°C	Nonpasteurized	65°C	75°C	Nonpasteurized	65°C	75°C
Pelargonidin 3‐rutinoside 5‐glucoside	1.18	1.01	1.05	1.42	0.41	0.44	0.80	0.64	0.47
Unidentified	—	—	0.31	—	—	—	—	—	—
Pelargonidin derivative	0.56	0.69	0.73	0.58	0.33	0.32	0.59	0.41	0.33
Unidentified	0.64	0.68	0.75	—	—	—	0.40	0.29	—
Pelargonidin 3‐coumaroylrutinoside 5‐glucoside isomer 1	0.54	0.69	0.74	0.56	0.42	0.44	0.41	0.68	0.37
Pelargonidin 3‐caffeoylrutinoside 5‐glucoside	0.92	0.78	1.23	1.05	0.53	0.58	0.64	0.75	0.53
Unidentified	0.86	0.60	—	1.40	0.46	—	0.38	—	—
Unidentified	—	—	—	—	—	—	0.29	—	—
Pelargonidin 3‐coumaroylrutinoside 5‐glucoside isomer 2	2.03	1.48	1.30	1.34	0.45	0.46	1.19	0.72	0.48
Pelargonidin 3‐coumaroylrutinoside 5‐glucoside isomer 3	17.23	13.99	17.69	16.07	5.01	5.70	7.73	9.40	5.71
Pelargonidin 3‐feruloylrutinoside 5‐glucoside	1.02	0.89	0.89	1.09	0.52	0.52	0.64	0.75	0.50
Cyanidin derivative isomer 1	—	—	—	0.60	—	—	—	—	—
Pelargonidin 3‐coumaroylrutinoside 5‐glucoside isomer 4	0.50	0.77	0.60	0.74	0.58	0.74	0,10	0.36	0.41
Pelargonidin 3‐coumaroylrutinoside 5‐glucoside isomer 5	0.28	0.43	0.42	—	—	—	0.93	0.75	0.74
Cyanidin derivative isomer 2	—	—	—	0.30	0.33	0.36	—	—	—
Unidentified	—	—	—	0.29	0.29	0.29	—	—	—

Many authors (Hernández‐Herrero & Frutos, 2011; Kırca et al., 2006; Mandha et al., 2023; Młynarczyk & Walkowiak‐Tomczak, 2017; Teleszko et al., 2016; Wang et al., 2012) prove that the stability of anthocyanins in fruit or vegetable juices depends on the type of raw material from which they are produced and the time, temperature, and technique of their thermal processing. Improving the stability of anthocyanins in fruit or vegetable juices subjected to pasteurization or other processes using high temperatures can be achieved by lowering the pH of the juices, for example, by acidifying them. This treatment may have a positive effect on the color of products and limit the degradation of biologically active compounds (Basílio et al., 2022; Gościnna et al., 2014; Li et al., 2013; Mgaya‐Kilima et al., 2014).

The pasteurization of potato juices at 65°C or 75°C for 5 min made their color lighter compared to the nonpasteurized juices (mean L* = 19.4) (Figures 1, 2). Smaller changes were observed in the juices pasteurized at 65°C for 5 min (mean L* = 18.3) (Figures 1, 2). Pasteurization increased the contribution of red (parameter a*) and blue (parameter b*) in the color profile of the analyzed juices (Figures 1, 2). Over time (after 4 h since juice extraction), the juices darkened and decrease was noted in the contribution of red and yellow colors in the juices made of both the red‐fleshed and the purple‐fleshed potato tubers. Greater changes in color parameters L*, a*, and b* were observed in the nonpasteurized juices.

The positive effect of temperature on the color of juices produced from purple‐fleshed potatoes or orange‐fleshed sweet potatoes was also demonstrated by Wang et al. (2012) and Eissa et al. (2021). Opposite results were reported by Rios‐Romero et al. (2021) who recorded lower values of L*, a*, and b* color parameters in the juices made of orange‐fleshed sweet potatoes after ultrasound and steam treatment. Alike findings were achieved by Aadil et al. (2013), Młynarczyk and Walkowiak‐Tomczak (2017), and Song et al. (2018) for juices made of grapefruits, elderberry, and maize. Pasteurization has a positive impact on the color of juices as it makes it lighter due to partial inactivation of polyphenolic oxidase and peroxidase. Its effects are largely determined by the raw materials the juices are made of as well as by chemical, physical, and biological reactions of their compounds. Heat treatment may also cause the juices to darken, mainly as a result of cavitation or degradation of color compounds present in the given raw material (Lee & Coates, 1999; Mandha et al., 2023; Wurlitzer et al., 2019; Vegara et al., 2013).

In addition, when analyzed immediately after pasteurization, the juices made of the colored‐fleshed potatoes had an increased chroma value (parameter C) and a decreased hue angle (parameter h°) compared to the nonpasteurized juices (Figure 3, Figure 4). After 4 h from the 5‐min pasteurization at 65°C or 75°C, the values of chroma decreased but the values of hue angle increased. The mean chromaticity of the juices made of the purple‐fleshed cultivars ranged from 28.20 (immediately after pasteurization) to 18.45 (4 h after pasteurization), whereas their mean hue angle ranged from 77.25 to 77.60, respectively. In the pasteurized juices extracted from the red‐fleshed potato cultivars, the mean C and h° values ranged from 54.45 to 46.60 and from 44.30 to 43.35, respectively. Also, Rios‐Romero et al. (2021) and Eissa et al. (2021) noted a decrease in both chroma value (parameter C) and hue angle (parameter h°) in the analyzed samples. The values of these parameters change in the juices upon heat treatment depending on the raw material they are made of (Mandha et al., 2023).

### 3.4 Principal components analysis (PCA)

PCA was conducted to summarize the appropriate grouping of all physicochemical parameters and properties linked to the potato juices (non‐pasteurized and pasteurized 65 or 75°C) obtained from potatoes of varieties with red or purple flesh (Figure 5). The first two main components explained 55.76% of the total variance (PC1 = 37.29% and PC2 = 18.48%). The first principal component was responsible for the differences between the color L*, a*, b*, C (after preparing potato juices, after 1 and 4 h), α‐solanine content (α‐s), content and composition of anthocyanis in purple of potato juices with Mal. derivative (P9), content and composition of anthocyanis in red of potato juices: Pel‐3‐rut‐5‐glu (R1), unidentified 1 (R2), Pel. derivative (R3), unidentified 2 (R4), Pel‐3‐coum‐rut‐5‐glu isomer1 (R5), Pel‐3‐caff‐rut‐5‐glu (R6), unidentified 3 (R7), Pel‐3‐coum‐rut‐5‐glu isomer 2 (R9), Pel‐3‐coum‐rut‐5‐glu isomer 3 (R10), Pel‐3‐feru‐rut‐5‐glu (R11), Cya. derivative isomer 1 (R12), Pel‐3‐coum‐rut‐5‐glu isomer 4 (R13), Pel‐3‐coum‐rut‐5‐glu isomer 5 (R14), Cya. derivative isomer 2 (R15), unidentified 5 (R16). PC2 was responsible for the differences between h° parameters (after preparing potato juices, after 1 and 4 h), total glycoalkaloids (TGA), total polyphenols content (TP), total anthocyanis content (TA), content and composition of anthocyanis in purple of potato juices: Pet‐3‐rut‐5‐glu (P1), Peo‐3‐rut‐5‐glu (P2), Del‐3‐coum‐rut‐5‐glu (P3), Pet‐3‐coum‐rut 5‐glu (P4), Pet‐3‐feru‐rut‐5‐glu (P5), Peo‐3‐couma‐rut‐5‐glu (P6), Peo‐3‐feru‐rut‐5‐glu (P7), unidentified (P8), Mal‐3‐coum‐rut‐5‐glu isomer 1 (P10), Mal‐3‐feru‐rut‐5‐glu (P11), Mal‐3‐coum‐rut‐5‐glu isomer 2 (P12), Pet‐3‐caff‐rut‐5‐glu (P13), Mal‐3‐coum‐rut‐5‐glu (P14), content and composition of anthocyanis in red of potato: unidentified 4 (R8). In addition, PCA showed differences as affected by variants of potato juices and the pasteurization process. The total polyphenols content (TP) in potato juices correlated positively with TA, h° parameters (after preparing potato juices, after 1 and 4 h), and composition of anthocyanins in the purple of potato juice: P4, P5, P10‐P14. Moreover, TA correlated with P1, P2, P4, P5, P10‐P12 and strong correlated with P13, P14. However, TGA was strongly correlated with α‐s, α‐ch, and composition of anthocyanins: P1, P3, P6‐P8. The color parameters L*, a*, b*, C (after preparing potato juices, after 1 and 4h) had a strong positive correlation with each other. The negative correlation observed between TP and α‐s, α‐ch, TGA, the composition of anthocyanins in the purple of potato juices: P1‐P3, P6‐P9, the composition of anthocyanins in red of potato juices: R2, R4, R7‐R10, R12, R15, R16 and R1, R3, R5, R6, R11, R13, R14 (strong negative correlation). The total anthocyanins content (TA) negatively correlated with α‐s, α‐ch, P3, P6‐P8, and all the composition of anthocyanins in the red of potato juices (R1‐R16). Moreover, TP and TA strongly negatively correlated with color parameters L*, a*, b*, C (after preparing potato juices, after 1 and 4 h). Additionally, TGA negative correlated with TA, TP, P4, P5, P10‐P14, R2, R14, R16. However, a negative correlation was observed between all the compositions of anthocyanins in the purple and red of potato juices.

## 4 CONCLUSIONS

The pasteurization process improved the color of potato juices from tubers of varieties with red and purple flesh. Potato juices pasteurized at 75°C were characterized by the lightest color and showed a higher share of red (increase in the value of parameter a*) and a higher share of yellow (increase in the value of parameter b*) compared to unpasteurized juices. The temperatures used in the juice pasteurization process also reduced the amount of glycoalkaloids by an average of 54% compared to unpasteurized juices; greater losses occurred in the content of α‐chaconine than α‐solanine. Potato juices differed in their composition and the amount of biologically active compounds determined. Juices obtained from purple potatoes had a higher content of total polyphenols and anthocyanins, on average 30% of total polyphenols and 70% of anthocyanins than juices from red potatoes. Among the anthocyanins identified in the juices of red potato varieties, pelargonidin and its derivatives dominated, while petunidin and peonidin dominated the purple ones. Higher losses of total polyphenols were found in juices from red‐fleshed tubers, while anthocyanins turned out to be less thermostable in juices made from purple‐fleshed potato varieties. The least thermostable were petunidin‐3‐feruloylrutinoside 5‐glucoside, peonidin 3‐coumaroylrutinoside 5‐glucoside, and peonidin 3‐feruloylrutinoside 5‐glucoside; pasteurized juices no longer contained these compounds.

## AUTHOR CONTRIBUTIONS

**Agnieszka Tkaczyńska:** Data curation (lead); formal analysis (supporting); investigation (lead); writing – original draft (supporting). **Elżbieta Rytel:** Conceptualization (lead); data curation (lead); project administration (lead); supervision (lead); writing – original draft (supporting). **Alicja Z. Kucharska:** Formal analysis (supporting); investigation (supporting); methodology (lead). **Joanna Kolniak‐Ostek:** Data curation (supporting); methodology (lead); software (supporting). **Anna Sokół‐łętowska:** Data curation (supporting); methodology (lead); software (supporting).

## FUNDING INFORMATION

The author obtained funds as part of the financing of a research project from National Science Centre, Poland, no.2019/35/O/NZ9/00168, entitled: The use of fruit and vegetable juices to stabilize the color of anthocyanins isolated from potatoes with purple and red flesh.

## CONFLICT OF INTEREST STATEMENT

The authors declare that they have no conflict of interest.

## Data Availability

The data that support the findings of this study are available on request from the corresponding author.

## APPENDIX A

**TABLE A1:** Dry matter (%) of potato varieties growing in season 2020/2021 and 2021/2022.

Potato varieties	Growing season 2020/2021	Growing season 2021/2022	Average
Provita	19.7	19.6	19.7
Double Fun	21.0	23.0	22.0
Violet Queen	20.2	21.5	20.9
Lily Rose	17.8	16.5	17.1
Magenta Love	16.8	17.8	17.3
Mulberry Beauty	20.1	23.0	21.6

**TABLE A2:** Dry matter (%) of the juice and juice after lyophilization (average of 2 years).

Variant	Variety of potato juice	Flesh color	Dry mass of juice	Lyophilisate
Nonpasteurized juice	Provita	Purple	5.41	90.85
65°C	Provita	Purple	4.57	90.82
75°C	Provita	Purple	4.26	89.43
Nonpasteurized juice	Double Fun	Purple	5.90	93.97
65°C	Double Fun	Purple	4.66	92.71
75°C	Double Fun	Purple	4.52	91.37
Nonpasteurized juice	Violet Queen	Purple	6.42	92.80
65°C	Violet Queen	Purple	5.91	91.63
75°C	Violet Queen	Purple	5.02	91.46
Nonpasteurized juice	Mulberry Beauty	Red	5.59	91.71
65°C	Mulberry Beauty	Red	4.59	88.80
75°C	Mulberry Beauty	Red	4.50	88.70
Nonpasteurized juice	Magenta Love	Red	4.72	91.52
65°C	Magenta Love	Red	4.17	86.14
75°C	Magenta Love	Red	4.43	85.71
Nonpasteurized juice	Lily Rose	Red	4.97	88.48
65°C	Lily Rose	Red	4.37	85.32
75°C	Lily Rose	Red	4.08	84.73

**TABLE 3A:** Color of non‐pasteurized potato juices immediately after pasteurization. 1 and 4 h after their pasteurization (average of 2 years).

		0 h					1 h					4 h				
Flesh color	Variant	L*	a*	b*	C	h°	L*	a*	b*	C	h°	L*	a*	b*	C	h°
Purple	Non‐pasteurized juice	0.33^a^	1.20^a^	0.30^a^	1.37^a^	185.5^d^	0.19^a^	0.71^a^	0.14^a^	0.83^a^	169.0^d^	0.16^a^	0.72^a^	0.07^a^	0.81^a^	176.4^c^
	65°C	8.31^b^	14.6^b^	10.7^b^	18.6^b^	77.1^c^	7.6^b^	14.3^b^	11.2^b^	18.6^b^	75.8^c^	6.5^b^	14.0^b^	10.0^b^	17.6^b^	77.9^b^
	75°C	8.33^b^	15.4^b^	10.4^b^	19.2^b^	74.0^c^	8.0^b^	14.8^b^	11.7^b^	19.4^b^	77.1^c^	7.5^b^	14.9^b^	11.4^b^	19.3^b^	77.3^b^
Red	Non‐pasteurized juice	0.69^a^	3.40^a^	1.04^a^	3.57^a^	34.9^a^	0.47^a^	2.50^a^	0.74^a^	2.62^a^	33.8^a^	0.33^a^	1.71^a^	0.46^a^	1.78^a^	43.4^a^
	65°C	28.2^c^	38.7^c^	38.0^c^	54.4^c^	44.4^b^	25.2^c^	37.0^c^	39.2^c^	54.2^c^	46.2^b^	18.3^c^	31.9^c^	29.8^c^	44.2^c^	41.4^a^
	75°C	30.5^c^	39.0^c^	37.8^c^	54.5^c^	44.2^b^	28.0^c^	37.5^c^	41.0^c^	55.9^c^	47.2^b^	22.0^d^	33.5^c^	35.2^d^	49.0^c^	45.3^a^
	LSD	3.26	4.15	4.24	5.69	9.31	3.09	4.07	4.65	5.86	12.4	2.79	3.66	4.85	5.49	31.9

*Note*: Data are expressed as the mean, *n* = 12. Results in the same column followed by different letters indicate significant differences according to Duncan's test at *p* < .05 between different flesh colors and variants, as determined by two‐way ANOVA.

Abbreviations: LSD, last significant difference.
